# Direct nano-imaging of light-matter interactions in nanoscale excitonic emitters

**DOI:** 10.1038/s41467-023-38189-y

**Published:** 2023-05-08

**Authors:** Kiyoung Jo, Emanuele Marino, Jason Lynch, Zhiqiao Jiang, Natalie Gogotsi, Thomas P. Darlington, Mohammad Soroush, P. James Schuck, Nicholas J. Borys, Christopher B. Murray, Deep Jariwala

**Affiliations:** 1 Department of Electrical and Systems Engineering, University of Pennsylvania, Philadelphia, PA 19104 USA; 2 Department of Chemistry, University of Pennsylvania, Philadelphia, PA 19104 USA; 3 Dipartimento di Fisica e Chimica, Università degli Studi di Palermo, Via Archirafi 36, 90123 Palermo, Italy; 4 Department of Materials Science and Engineering, University of Pennsylvania, Philadelphia, PA 19104 USA; 5 Department of Mechanical Engineering, Columbia University, New York, NY 10027 USA; 6 Departement of Physics, Montana State University, Bozeman, MT 59717 USA

**Keywords:** Scanning probe microscopy, Quantum dots, Nanophotonics and plasmonics

## Abstract

Strong light-matter interactions in localized nano-emitters placed near metallic mirrors have been widely reported via spectroscopic studies in the optical far-field. Here, we report a near-field nano-spectroscopic study of localized nanoscale emitters on a flat Au substrate. Using quasi 2-dimensional CdSe/Cd_x_Zn_1-x_S nanoplatelets, we observe directional propagation on the Au substrate of surface plasmon polaritons launched from the excitons of the nanoplatelets as wave-like fringe patterns in the near-field photoluminescence maps. These fringe patterns were confirmed via extensive electromagnetic wave simulations to be standing-waves formed between the tip and the edge-up assembled nano-emitters on the substrate plane. We further report that both light confinement and in-plane emission can be engineered by tuning the surrounding dielectric environment of the nanoplatelets. Our results lead to renewed understanding of in-plane, near-field electromagnetic signal transduction from the localized nano-emitters with profound implications in nano and quantum photonics as well as resonant optoelectronics.

## Abstract (web summary)

Authors investigate quasi-2D nanoscale emitters on different substrates with tapping mode tip-enhanced spectroscopy. They visualize in-plane near-field and radiative energy propagation via Surface plasmon polaritons launched by the nanoscale emitters on dielectric/Au or SiO_2_/Si substrates.

## Introduction

Understanding of light-matter interactions in materials with strongly resonant properties and deep-subwavelength dimensions is important for both basic science and nano-opto-electronic applications. In most cases, light does not modify the electronic dispersions of the materials. This is because either the material dimensions are much greater than the wavelength λ or the material does not have an electronic dipole resonance at λ resulting in weak coupling. When the coupling between light and dipoles in matter becomes stronger, the rapid exchange of energy between photon states and electric dipole resonances leads to the formation of part-light, part-matter quasiparticle states called polaritons^1^. Different types of dipoles form different types of polaritons, including plasmon-polaritons in metals, exciton-polaritons in semiconductors, and phonon-polaritons in dielectrics in the IR range^2–5^. Such strong light-matter couplings require a confinement of light in a low-dimensional material or interface to efficiently interact with the dipoles. For the case of surface plasmon polaritons (SPPs), light is confined to a dielectric/metal interface and forms a propagating electromagnetic wave along the surface^6^. Conversely, the formation of exciton-polaritons requires an intrinsic optical resonance of the medium to overlap with trapped light wave-packets in an optical cavity medium such as Bragg mirror dielectric microcavity or plasmonic cavity^1, 5^. Propagating modes of exciton-polaritons were also observed with help of scattering type nanoprobes^7,8^ and microcavities^9,10^.

In reduced dimensional materials, the exciton that is induced by light-excitation interacts strongly with the surrounding medium due to the lack of dielectric screening. The binding energy of the excitons in 2-dimensional (2D) WSe_2_ (0.78 eV)^11^, 1-dimensional (1D) single-walled carbon nanotubes (0.3–0.4 eV)^12^ and 0-dimensional (0D) CdSe quantum dots (0.2–0.8 eV)^13^ are significantly larger than room temperature thermal energy (0.025 eV). The large exciton binding energy in these nanomaterials makes excitons – not free carriers - the dominant excited species, resulting in stronger light-matter interaction. Strong light-matter coupling in excitonic nanomaterials has been investigated in many ways such as exciton-polaritons in 2D MoS_2_ placed in an optical cavity^14^, exciton-plasmon polaritons of 2D WSe_2_^15^, 0D CdSe/ZnS quantum dot placed in plasmonic cavities^16,17^, and surface plasmon polaritons at 2D MoS_2_/Al_2_O_3_/Au interfaces^18^. Most of these studies have been conducted in either diffraction-limited optical setups^14–16^ or via non-optical excitation techniques such as electron energy loss spectroscopy^19^. However, imaging of strong light-matter coupling in nanoscale materials when excited in the near-field at optical frequencies^17,20^ has been scarcely investigated. Further, the impact of the nano-probe and complex nano-optical fields on dipole interactions as well as energy guiding and transduction at these deep sub-wavelength scales remains largely unexplored.

Tip-enhanced nano-spectroscopy has paved the way for direct nano-resolution spatio-spectral imaging of the emission of nanomaterials at optical frequencies^1,21^. By taking advantage of plasmonic gap mode confined in the nano-gap between the plasmonic tip and the substrate, this technique has enabled the visualization of optical responses from sub-wavelength semiconductor structures such as fluorescence/radiation patterns of quantum dots^20^, strain-induced Raman and fluorescence shifts^22,23^, lateral heterostructures of van der Waals semiconductors^24,25^, and even the localized excitonic emission from nanobubbles in 2D semiconductors^26^. Most studies of the emission using tip-enhanced nano-spectroscopy are performed in contact mode to maximize the plasmonic gap mode confinement resulting in strong light-matter coupling along the normal direction to the surface. For example, strong light-matter interactions in CdSe/ZnS quantum dots using the plasmonic gap mode was achieved leading to exciton-plasmon polariton formation^27^. In addition, brightening of the dark exciton in monolayer transition metal dichalcogenides via the Purcell effect has also been observed^28^. Yet, investigations for inelastic emission or scattering with the tapping mode configuration have been limited^29,30^. Since the AFM tip oscillates in tapping mode, it is still close to the surface hence it can be considered a near-field signal. Recently, it was reported that tapping mode, tip-enhanced Raman maintains sub-wavelength resolution capability and is even beneficial in terms of a charging-free measurement tool by preventing hot-carrier injection^31,32^. However, the role of the tapping mode tip in emission remains elusive.

Herein, we report in-plane light-matter interactions of quasi-2D emitters on dielectric/Au or SiO_2_/Si substrates with tapping mode tip-enhanced spectroscopy. When using tapping mode tip-enhanced spectroscopy, we visualize in-plane near-field radiation and radiative energy propagation via SPPs launched by the emitters on dielectric/Au or SiO_2_/Si substrates. By placing the nanoscale emitters, such as CdSe/Cd_x_Zn_1-x_S nanoplatelets and WSe_2_ nanobubbles, on dielectric/Au interfaces, we observe radiative fringe patterns that are indicative of sub-wavelength energy transfer from the nanoscale excitonic emitters to the plasmonic Au substrate. We further observe that dielectric permittivity and thickness are key parameters that control the observed fringe patterns and corresponding energy transfer. Our results facilitate a deeper understanding of near-field radiation from low-dimensional and hetero-dimensional excitonic systems.

## Results

### In-plane surface plasmon polariton launched by emission from nanoplatelets at a dielectric/Au interface

**Fig. 1:**
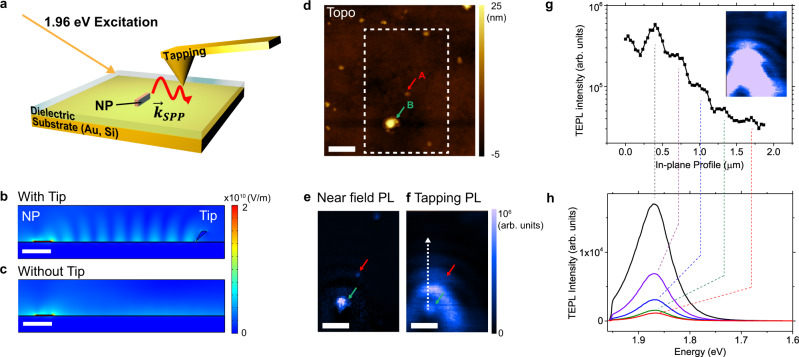
Characterization of nano-optical fringes. **a** Schematic representation of the surface plasmon polariton launched by NP agglomerates and collected by the Au tip working in tapping mode for a dielectric-NP on Au. **b**, **c** 2D map of the simulated E-field strength for Al_2_O_3_ (5 nm)/NP/Au at 1.86 eV (664 nm wavelength) with and without the Au tip. **d** Topography image of Al_2_O_3_ (5 nm)/NP/Au and **e** corresponding hyperspectral tip-enhanced photoluminescence (TEPL) map of the dashed region of (**d**) with near-field TEPL (contact mode TEPL subtracted by tapping mode TEPL) and **f** tapping mode TEPL (1.86 eV) of NP cluster and exciton-launched SPP scattered by the tip at dielectric/Au interface. **g** Photoluminescence fringe intensity profile along the dotted arrow line in (**f**). Inset: tapping mode TEPL map in (**f**) with a saturated intensity scale. **h** the corresponding photoluminescence spectra. Scale bars indicate 0.5 μm. The red and green arrows at (**d**, **e**, **f**) represent the NPs with face-down (red) and edge-up (green) configuration.

We investigate the evidence of exciting in-plane polariton with quasi-2D CdSe/Cd_x_Zn_1-x_S core-shell nanoplatelets (NPs) on ultrasmooth Au substrate. Using TEM, we measure the size of the NPs to be 40.2 ± 2.9 nm (length) × 16.1 ± 1.7 nm (width) × 2.8 ± 0.5 nm (thickness). To collect the in-plane near-field signal from NPs, we obtained hyperspectral map using tapping mode operation (Fig. S1 for the details). We note that tip under contact mode leads to different type of strong light-matter coupling (See Analyst section in SI and Fig. S2). In addition, there is no charge-induced quenching due to the proximate Au substrate (see Fig. S3). Since the Au tip serves as a scatterer in the near-field, this allows for the collection of light propagating along a dielectric/metal interface (Fig. 1a). At the dielectric/metal interface, light is confined to the interface and propagates along the surface by forming surface plasmon polaritons (SPPs). In the dielectric/NP/Au system that we are investigating, NPs that are hundreds of microns away from the tip can be excited by a laser or by a SPP launched by the tip toward the NPs as reported in various studies using a scattering-type tip^33–36^. The emission from the NPs launches successive SPPs along the high index dielectric/metal interface and directly interferes with itself upon reflection from a distant Au tip. This observation is consistent with previous reports on SPPs launched by nano-emitters in the vicinity of the plasmonic graphene sheets^37,38^. For the SPP excitation from NPs, we speculate that the SPP can be coherently excited by excitonic photoluminescence because exciton coherence dephasing time of the NPs at room temperature (~10–25 fs)^39^ is comparable to the dephasing time of the SPP at Al_2_O_3_/Au interface (~10 fs)^40^. As a result, the tip reflects the SPPs back to the emitter forming a standing wave. Standing wave formation have been observed in different types of polaritons via scattering mode n-SOM i.e. near-IR SPP in graphene^33,34,41^, exciton-polaritons in bulk WSe_2_^35^, and phonon-polaritons in hBN metasurfaces^2,36,42^. In these previous reports, the tip, in proximity of specimen launches the polariton. On the other hand, in our present work, both NPs and the tip can launch the SPPs along the dielectric/Au interface. To evaluate the contribution of each case, we simulated the cross-sectional E-field profile of a dielectric/NP/Au structure in the vicinity of the Au tip engaged in tapping mode, i.e. 20 nm away from the Au surface, to visualize the generation of these standing waves. Figures 1b and c verifies that the fringes only appear when both the NP and tip are present and close to the Au substrate which supports our explanation that the tip reflects the SPP and forms fringe patterns. As the tip-NP distance increases, the E-field intensity periodically changes, proving the standing wave condition changes with tip-NP distance (Fig. S4). Moreover, a comparison of the E_z_ field map with and without the tip demonstrates that surface-confined electric field persists from SPPs regardless of the presence of tip (Fig. S5). This is also consistent with our explanation that the excitonic emitter launches SPPs as previously reported in graphene plasmons induced by nanoemitters^37,38^. Yet, the tip could be either a reflector of SPPs launched by NPs or a SPP source launched by incident light. We evaluated the contribution of the tip plasmon toward the formation of the fringes by simulating the system without a NP (Fig. S6b). In this simulation, we placed a dipole emitter far away from the Au substrate, but the tip is kept close to the substrate. We found that the tip launches SPPs (Fig. S6b, d) though its strength is 20-100 times smaller than the fringes found in NP-tip system. We note that the quantitative analysis may not be valid, and the tip-induced SPP is comparable to SPP launched by NP when the nanoemitter is not highly emissive i.e. off-resonant excitation, low quantum yield nanoemitter. In nearly on-resonant excitation of the exciton, it is hard to distinguish SPPs launched by the NP vs the tip. Therefore, we can generalize the mechanism of fringe formation as one achieved by interference between SPPs launched by tip and NP.

With this mechanism, a standing wave is expected to form between the NP and the Au tip with the following condition,

1 $$\frac{{\lambda }_{{SPP}}}{2}N={L}_{n}$$

where λ_SPP_ is the wavelength of the SPP, *N* is an integer and *L*_*n*_ is the length of cavity which is determined by the distance between the tip and the emitter.

Figure 1b shows the multiple fringes emerging between the NP and the Au tip which we refer to as a SPP standing wave. The Fourier transform of the fringe pattern yields a period of 317 nm, which is consistent with the expected standing wave SPP period $\frac{{\lambda }_{{SPP}}}{2}$ = 320 nm. The expected period is calculated according to the dispersion relation of SPPs,

2 $${\lambda }_{{SPP}}={\lambda }_{o}\sqrt{\frac{{\epsilon }_{d}+{\epsilon }_{m}}{{\epsilon }_{d}{\epsilon }_{m}}}$$

where λ_SPP_ and λ_o_ are the wavelengths of SPP and photoluminescence (PL) emission, respectively and ${\epsilon }_{d}$ and ${\epsilon }_{m}$ are dielectric constants of the dielectric and metal, respectively.

The simulations presented in Fig. 1b indicate that the Au tip acts as a reflector for SPPs, and that the standing wave condition can be controlled by changing the distance between the NP and Au tip. To verify the interference pattern, we obtained hyperspectral tip-enhanced photoluminescence (TEPL) maps with ~20 nm spatial resolution of an Al_2_O_3_ (5 nm)/NP/Au system. The topography and corresponding hyperspectral TEPL maps were obtained with tapping mode during the same scan (Fig. 1d–f) and can therefore be used for fair comparison. NPs are quasi-2D nanocrystals which support excitons with dipoles oriented along the crystal plane. However, due to their relatively small aspect ratio (NP width/thickness ~14) compared to 2D materials (ideally infinite), either the edge or plane of the NP is in contact to the substrate which leads to different dipole orientation with respect to the substrate (Fig. S7b). It has been reported that the transition dipole orientation of edge-up assembled NP (edge of NP is in contact with substrate) is perpendicular whereas the dipole orientation of face-down assembled counterparts (NP plane is in contact with substrate) is parallel with respect to the substrate^43^. The NP clusters A and B in Fig. 1d represent NPs with face-down (A) and edge-up (B) configuration (Fig. S7c). Considering the size of a single NP plate (40 × 16 × 2.8 nm^3^), region A was determined to be a face-down configuration by its thickness (12 nm) which corresponds to the thickness of 2 NPs surrounded by 2 nm oleate ligand layer. Cluster B was characterized as edge-up configuration with 2 NPs with ligands which coincides with 40 nm thickness. Near-field TEPL of the two different regions shows spatially localized emission at 664 nm (Fig. 1e). In contrast, a boomerang-shaped fringe pattern arises from the NP at spot B at 664 nm when measured in tapping mode (Fig. 1f, h) while the NP at spot A does not. The fringes do not appear in far-field PL map, indicating the fringe is near-field phenomenon (Fig. S7d, e). This source-selective fringe pattern can be explained by the different transition dipole orientation of A and B which we will explain in the following section. Note that the thickness of NPs also affects the E-field intensity (Fig. S8). Quantitatively, a 9-fold enhancement of the E-field with 20 nm thick NPs was obtained as compared to 2.8 nm thin NP. However, the fringe pattern appears even in the 2.8 nm case, which supports our claim that the exciton dipole orientation in NPs predominantly determines the fringe formation from the exciton-launched SPPs. The comparison between TEPL under different modes demonstrates that the tapping mode does not affect SPP fringe formation, but rather serves as an antenna which scatters and aids collection of the propagating SPP launched from the excitons in the NPs. Therefore, it is necessary to distinguish the terminology for the emission at the NP location and the radiative fringes formed at the dielectric/Au interface away from the NPs. The former represents TEPL directly from NPs while the latter stands for the emission from SPPs launched by the excitons in NPs that are scattered into the far-field by the tip, hereby referred to as exciton-launched SPPs. Fourier analysis of the fringe pattern (see SI for details, Fig. S9) reveals that the fringe period in the boomerang-shaped pattern is 322 ± 119 nm at the symmetry axis of the boomerang (arrow line in Fig. 1f) which is similar to the simulated fringe period (Fig. 1b, g). Note that the error is measured using the full width at half maximum (FWHM) of the peak appearing in the Fourier transform. The large error bars are due to the lossy plasmonic cavity of dielectric/metal medium. The similarity in fringe periods between the experiment and the simulation suggests that SPP standing waves are responsible for producing the boomerang-shaped fringe patterns in near-field emission maps. SPPs in the near-field are usually measured in scattering mode using dark-field imaging to collect the scattered light that has propagation directions that are highly misaligned from the normal direction^44^. Here, the scattered SPPs are measured in emission mode since the SPPs are launched from the excitonic emission of highly emissive NPs and the tip is close enough to the interface to allow collection of the scattered light from the interface. As the tip scans the sample close to an excitonic emitter, it encounters standing wave conditions at specific locations and collects all the scattered light by the tapping mode tip. This indicates that the tapping mode maintains sub-wavelength resolution. Hence, the tapping mode TEPL map is not just a far-field spectrum, but it is a direct imaging tool for in-plane light propagation. Further, the fringes in emission mode are valuable in investigating the contributions of different exciton dipole orientations on the excitation of SPP which we cover in the next section. The boomerang-shaped fringes appeared until the 5th fringe ~ 1.7 μm away from the source with the intensity of emission from scattered SPPs diminishing below detection levels beyond that, as shown in Fig. 1g, h. The exciton-launched SPP intensity at the antinode was fitted with an exponential decay curve, revealing a decay constant of 324 ± 0.01 nm (Fig. S10a). Since the tip was away from the NP cluster, the interference intensity decreases due to the lossy cavity of the dielectric/Au interface.

### Geometric configuration and polarization dependent standing wave surface plasmon polaritons

**Fig. 2:**
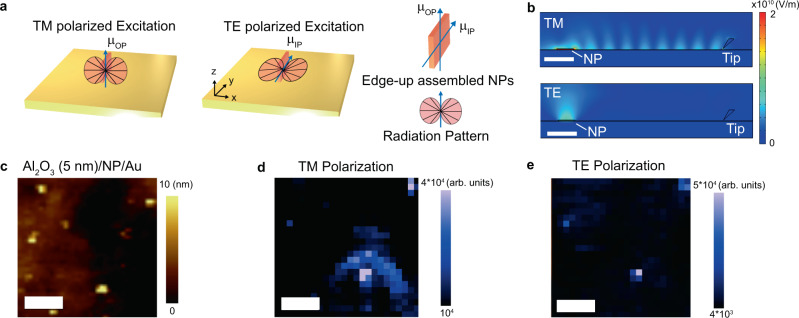
Nano-optical fringes with respect to exciton transition dipole orientations. **a** Schematic representation of the radiation pattern from dielectric/edge-up assembled NP/Au system with Au tip under transverse magnetic (TM) polarization (left) and transverse electric (TE) polarization (middle). **b** Simulated identical system under TM polarization (top) and TE polarization (bottom). **c** AFM topography image of Al_2_O_3_ (5 nm)/NP/Au and hyperspectral tapping mode TEPL under **d** TM polarization and **e** TE polarization. Scale bars indicate 0.5 μm.

The coupling between the PL emission from the NPs and the plasmons depends on the polarization of the fields of the emitted light and the excitation laser. For quasi-2D CdSe/Cd_x_Zn_1-x_S NPs, the degenerate transition dipole is oriented along the plane of the NP^45–47^. This leads to polarized and directional emission perpendicular to the major plane of NP, which has been previously reported and analyzed by the Hertzian model and also accounting for the anisotropic dipole orientation^48^. Therefore the geometric configuration of the NP, i.e. edge-up and face-down assembled NPs (Fig. S7b), determines the polarization of emission as well as strength of coupling with the plasmons. In the as-prepared sample, edge-up assembled NP clusters can form a dominant out-of-plane vs in-plane excitonic transition dipole with respect to TM vs TE polarized excitation, leading to different types of directional emission (Fig. 2a). Because the exciton dipole orientation under TM polarized excitation is parallel to the field orientation of surface plasmon, it results in strong coupling, and therefore, the edge-up assembled NPs can launch the SPP^43^. Our finite element simulations support our explanation that TM polarized emission launches the SPPs (Fig. 2b, top). On the other hand, both edge-up and face-down assembled NP clusters forms dominant in-plane oriented excitons with transverse electric field (TE). Since the exciton transition dipole orientation is perpendicular to field direction of the surface plasmon, it can neither couple with the plasmon nor launch the SPPs (Fig. 2b, bottom).

**Fig. 3:**
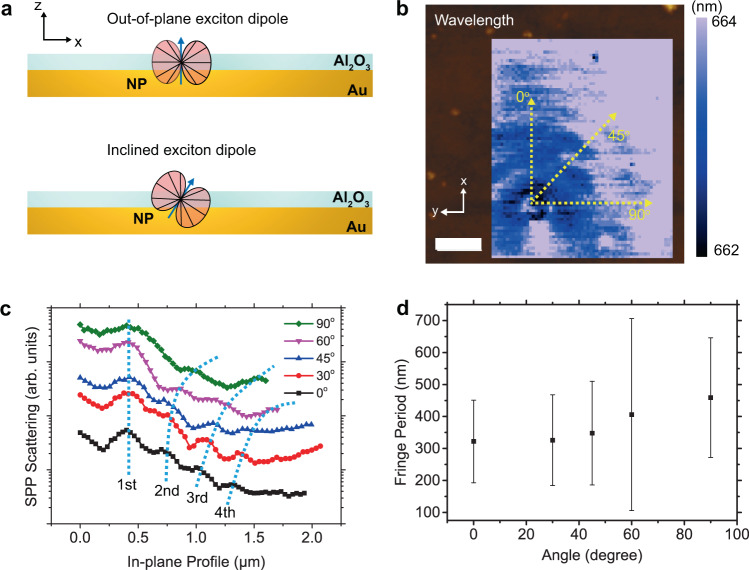
Analysis of fringe patterns. **a** Schematic representation of exciton transition dipole oriented out-of-plane (top) and at an inclined angle (bottom). **b** Near-field emission wavelength map of Al_2_O_3_ (5 nm)/NP/Au with topography background and **c** TEPL intensity line-cuts at different direction angles. Dotted lines are drawn as a guide for the eye. **d** Fringe period as a function of angle. Error bar is FWHM of the peak appearing in the Fourier transform of fringe profiles in (**c**). Scale bar indicates 0.5 μm.

Considering the polarization of the PL emission from NP clusters and the excitation laser, only the combination of TM polarized light and the edge-up assembled NP cluster can launch SPPs. To verify the simulations with experiments, we conducted a polarization-dependent exciton-launched SPPs map to evaluate the relationship between the excitonic dipole and fringe formation (Fig. 2c–e). The exciton-launched SPP response differs with respect to the polarization of the excitation laser. Fringe patterns are observed for TM polarization at the edge-up assembled NP clusters, agreeing with our hypothesis that the out-of-plane transition dipoles excited by the TM polarized laser emits in-plane directional light, successively launching an SPP (Fig. 2b, d). On the other hand, TE polarized light excites excitons with in-plane transition dipoles, leading to negligible in-plane SPP generation (Fig. 2b, e). We further note that fringe patterns only appear for thick NP clusters (>16 nm) whereas they do not appear for thin NP clusters (~3 nm). This observation again coincides with our claims that the edge-up NP clusters launch SPPs while the other do not. That is, we can switch the SPPs launched from individual edge-up assembled NP clusters by modulating the polarization of the excitation laser, which was directly observed in our experiments (Fig. 3).

### Fringe shape and directionality of the generated surface plasmon polariton

If the transition dipoles of the NPs are oriented normal to the substrate (Fig. 3a), the fringe pattern would be circular because the SPPs would be launched uniformly in all in-plane directions. However, the fringe pattern observed in the exciton-launched SPP map (Fig. 1f) has a parabolic shape which reveals that the transition dipole orientation in the NPs is not normal but inclined. This claim is supported by the 3D finite element simulation that an out-of-plane dipole (Fig. S11c) launches circular fringes while an inclined dipole (Fig. S11d) creates parabolic or boomerang-shaped fringes due to the broken symmetry. The dependence of the shape of the fringe pattern on exciton dipole orientation is significant since it allows experimental probing of dipole orientations in nanostructured emitters which is non-trivial. Further, fringe patterns appear only at the lateral cavity between NPs and tip (Fig. S11c, d). The fringe disappears outside the cavity revealing the directionality of the fringes is determined by the tip displacement. Therefore, the transition dipole orientation and tip displacement during the scan matters in determination of the shape of fringes. To experimentally support this, we analyzed azimuthal angle dependent fringe period (Fig. 3b). The background in Fig. 3b shows the exciton-launched SPP wavelength map of identical Al_2_O_3_/NP/Au system previously shown in Fig. 1. All the fringes have similar peak wavelengths that are close to the excitonic emission wavelength of the NP. The negligible energy shift means that SPPs that are generated by NP clusters propagates until they are reflected or scattered by tip. There is negligible signal detected at non-excitonic wavelengths (Fig. S12c). We, therefore, ensure that the SPP launched from the NPs is mainly responsible for the fringe shape. The boomerang-shaped fringe indicates that the dipole orientation is not perfectly out-of-plane but inclined based on our simulations (Fig. S11c, d). The exciton-launched SPP intensity profiles along lines oriented from 0 to 90 degrees with respect to the symmetry axis were investigated to understand the directionality of the NP PL emission (Fig. 3c, d). The fringe period is observed to increase with increasing angle, eventually disappearing. Quantitatively, the fringe period is 322 nm at 0 degrees whereas it is 459 nm at 90 degrees. This difference suggests that interference does not occur at a high angle due to lack of light directly heading to the tip and therefore, the NP-tip cavity does not form.

### Direct observation of dielectric effect on the surface plasmon polariton

**Fig. 4:**
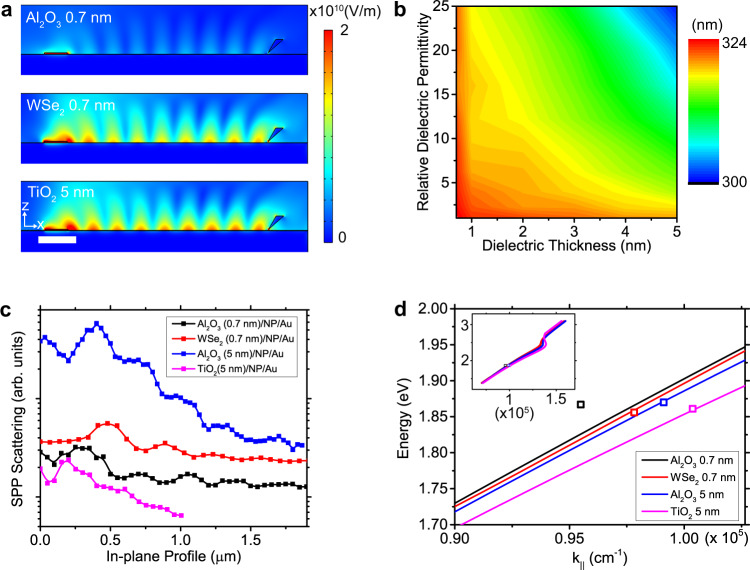
Direct observation of dielectric effect on the SPP. **a** 2D map of the simulated E-field intensity (4500 nm width × 600 nm height) with different dielectric layers. **b** Simulated fringe period as a function of relative dielectric permittivity (*ε*) and thickness of the dielectric layer. **c** Exciton-launched SPP intensity line cuts in hyperspectral images along with x direction. **d** Simulated E-K diagram of SPPs at a dielectric/Au interface (line) and experimental results (dots). Inset is the full range E-K diagram. Scale bar in (**a**) indicates 0.5 μm.

Since a SPP is a surface-confined electromagnetic mode, it is subject to alterations by changes in the dielectric medium at the interface. It is possible to observe these effects as small perturbations of the fringe pattern as the exciton-launched SPP scattered by tip under tapping mode operation reports the intensity distribution of the near-field electromagnetic wave bound to the dielectric/metal interface. Therefore, we investigate the variation of the fringe pattern as a function of the permittivity and thickness of the dielectric. Simulations were performed using different dielectric materials and thicknesses. We used dielectrics that are readily deposited in thin uniform films via ALD or mechanical stamping such as Al_2_O_3_ (ε = 3.13 at 630 nm), TiO_2_ (ε = 5.02 at 630 nm) and monolayer WSe_2_ (ε = 15 at 630 nm). A 0.7 nm thick Al_2_O_3_ sample was prepared to compare with monolayer WSe_2_. Figure 4a shows the simulated standing wave fringe patterns with different dielectric configurations. These results demonstrate that a larger difference in *ε* between the dielectric material and metal creates a stronger confinement of electromagnetic waves at the interface. To generalize the dielectric effects on fringe periods, we simulated fringe patterns as a function of dielectric constant and thickness that is summarized in the 2D plot in Fig. 4b. The period of the simulated standing wave increases with decreasing dielectric permittivity and dielectric thickness. We deposited Al_2_O_3_, TiO_2_ and monolayer WSe_2_ on as-prepared NPs on the Au substrate to verify experimentally the validity of the simulations. Note that the thickness of the NP cluster is ~30 nm for all samples and the emission spectrum peaks at ~1.85 eV (664 nm, i.e. the excitonic wavelength) for all samples. Similar boomerang patterns were observed in Al_2_O_3_ (0.7 nm), WSe_2_ (0.7 nm) and TiO_2_ (5 nm)/NP/Au samples (Fig. S13a–c). However, distinct differences were observed in their periods (Fig. 4c). This observation shows that even the thinnest dielectric confinement can modify the SPP propagation which can be recorded via the interference effect using a reflective tip. Compared to the fringe period (322 nm) of Al_2_O_3_ (5 nm)/NP/Au system, we observe 329 ± 135 nm for Al_2_O_3_ (0.7 nm), 325 ± 119 nm for WSe_2_ (0.7 nm) and 313±92 nm for TiO_2_ (5 nm)/NP/Au, respectively. Once again, the large error bars are due to lossy plasmonic propagation. The E-K diagram of SPPs generated at the dielectric/Au interface was simulated by adopting Lorentz-Drude model for Au (see Analysis section in SI)^49^. The experimental values are in close agreement with the simulated E-K diagram (Fig. 4d). The experimental value from Al_2_O_3_ (0.7 nm)/Au interface deviates from the calculated E-K diagram, possibly due to a non-uniform dielectric medium for <1 nm thin ALD grown film of alumina. The strong agreement between calculations and experiments reveals that the small variation of SPP due to dielectric change can be detected by near-field scanning probe technique. It also excludes the possibility that the fringe is the result of diffraction, as the diffraction of free-space light cannot be affected by a deep-sub-wavelength thickness dielectric layer. The SPP decay constant, which directly correlates to energy transfer, shows the opposite relation with the dielectric constant of the medium i.e. larger *ε* results in longer decay constant. Quantitatively, high dielectric materials such as TiO_2_ and WSe_2_ showed decay constants of 468 ± 0.16 nm and 417 ± 0.12 nm respectively (Fig. S10c, d). This result coincides with our simulations (Fig. 4a) showing that high dielectric medium results in high E-field strength of the SPP due to stronger confinement. The comparison between two different thickness of Al_2_O_3_ layers (324 ± 0.11 nm for 5 nm Al_2_O_3_ and 234 ± 0.12 nm for 0.7 nm Al_2_O_3_) shows that the thicker dielectric medium is more effective for in-plane light propagation (Fig. S10e).

### Universal emergence of fringe pattern by varying emitter or interface property

**Fig. 5:**
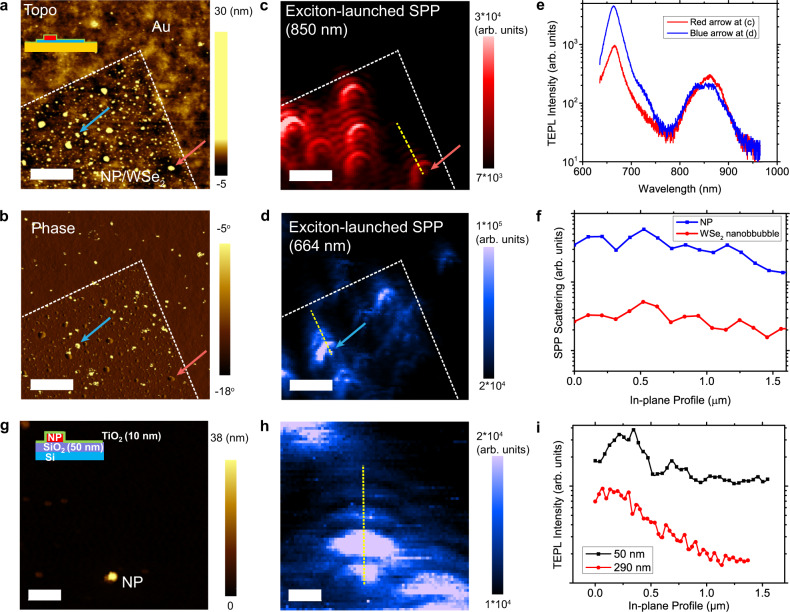
Universality of nano-optical fringes. **a** Topography and **b** Phase map of Al_2_O_3_(0.7 nm)/NP/WSe_2_/Au **c**, **d** Hyperspectral mapping of the exciton-launched SPP at WSe_2_ nanobubble emission wavelength and NP emission wavelength. **e** Exciton-launched SPP spectrum at red arrow in (**c**) (WSe_2_ bubble) and blue arrow in (**d**) (NP). **f** Exciton-launched SPP intensity line cuts along the yellow dotted lines in (**c**) and (**d**). **g** Topography of TiO_2_(10 nm)/NP/SiO_2_(50 nm)/Si and **h** hyperspectral tapping mode TEPL map at NP emission wavelength (**i**) TEPL intensity line cuts along yellow dotted line in (**h**). The black curve is for a 50 nm SiO_2_ layer and the red is for 290 nm SiO_2_. Scale bars in (**a**–**d**) indicate 2 μm. Scale bars in (**g**, **h**) indicate 0.5 μm. All measurements are conducted with 633 nm excitation laser.

To generalize this fringe phenomenon to other localized emitters, we explore the same effect in nanobubbles of monolayer WSe_2_. Monolayer WSe_2_ was prepared using mechanical exfoliation on a template-stripped Au substrate. To directly compare the fringe patterns produced by NPs to those of nanobubbles, we spin-coated NPs onto the exfoliated WSe_2_ and then deposited a thin film of Al_2_O_3_. The ALD of the Al_2_O_3_ film led to an ensemble of nanobubbles with emission centered at 850 nm. The large redshift of the excitonic emission can be attributed to strain-induced bandgap lowering^26^. The monolayer WSe_2_ flake contained nanobubbles that spanned 100 nm in diameter (Fig. 5a). The phase map clearly distinguishes the NPs from nanobubbles (Fig. 5b). NPs show an abrupt contrast change (blue arrow) whereas WSe_2_ nanobubbles do not (red arrow). WSe_2_ bubbles which confine excitons via spatial modulation of band structure due to strain also act as localized emitters^50^ which emit at 850 nm and launch fringes with 357 ± 163 nm period (Fig. 5c). We also investigated SPP fringes launched by excitons in WSe_2_ nanobubbles with different excitation energies i.e. 594 nm (2.08 eV) and 785 nm (1.58 eV). The results revealed that the fringe period under 594 nm excitation was similar to those launched with 633 nm excitation, while the fringe period under 785 nm excitation was longer (Fig. S14f). We attribute this to the nano-emitter acting as a scatterer of the incident laser, resulting in another type of SPP launched via laser scattering, hereby referred to as laser-scattered SPP. In principle, the energy of a laser-scattered SPP is identical to the energy of laser due to elastic nature of Rayleigh or Mie scattering. Consequently, it cannot be detected by our measurement since we mount an edge filter for the excitation laser on the collection side of our apparatus. Nevertheless, the laser-scattered SPP can broaden in energy due to ohmic losses as it propagates in the Au. This results in a broad shoulder being captured in our hyperspectral map if the incident laser energy is close to emission spectral window. Therefore, in the map of fringes observed under different laser excitation energies, the exciton-launched SPPs are the dominant cause of fringes for 594 and 633 nm excitation whereas the laser-scattered SPPs may be significantly contributing to the fringes observed under 785 nm excitation. To further verify our experimental observations, we simulated SPPs launched by a 790 nm excitonic dipole with a WSe_2_ layer on Au and observe a fringe period of 380 nm (Fig. S14f), which is in good agreement with the experimental results. This verifies that exciton-launched SPP from WSe_2_ nanobubbles can be accurately visualized under 594 and 633 nm excitation, by minimizing the impact of laser-scattered SPPs. These observations suggest that excitation laser energy should be far from excitonic emission to collect exciton-launched SPPs from nano emitters. In our observation of SPPs from NPs, as discussed in Figs. 1–4 above, we employ a 1.96 eV excitation laser while the excitonic emission was at 1.85 eV. This also explains the strong agreement between our experiments and simulations in Fig. 4d above. Meanwhile, the fringe period of NPs with 633 nm excitation laser was 322 ± 128 nm on the same substrate which is smaller than that of WSe_2_ nanobubbles (Fig. 5d). This is primarily because the wavelength of excitonic emission is more red-shifted for WSe_2_ nanobubbles which requires a longer path length to attain the standing wave condition. Moreover, fringe shape of WSe_2_ nanobubbles was circular which means its transition dipole orientation is nearly normal to the surface. On the other hand, the boomerang-shaped fringes are launched by the NP clusters due to inclined dipole. To further investigate the effect of NP-tip cavity to form fringes, we rotate the sample and measured exciton-launched SPP again (Fig. S15). We observed same directional fringe formation after 90 degree sample rotation that again verifies that the standing wave is originated from NP - tip lateral cavity. In addition, the nanobubbles of different 2D semiconductor, WS_2_, also launched fringes due to the SPP at 660 nm (Fig. S16). This experiment and observation confirm that the localized excitons emitting at different emitting frequencies are all capable of launching SPPs on plasmonic substrates, which can be observed in near-field in the form of fringe patterns with varying periods.

Finally, we also verify that the in-plane optical propagation of excitonic emission by localized emitters can lead to the formation of fringe patterns even in the case of non-plasmonic (dielectric) substrates. In this case the emitted light is expected to be confined into a waveguide mode that propagates and is reflected back from Au tip forming fringe patterns representing standing waves. To investigate this in-plane guided-mode light propagation in a dielectric layer, we fabricated TiO_2_ (10 nm)-NP structures on SiO_2_ (50 nm)/Si substrates rather than plasmonic Au substrates (Fig. 5g). Note that TiO_2_ is pre-selected for a higher refractive index (2.24) than that of the SiO_2_ (1.46) to ensure larger light confinement in the SiO_2_ layer. We observed a similar fringe pattern of the excitonic emission at 663 nm with a 50 nm thick SiO_2_ layer (Figs. 5h, i and S17c–f). This supports our hypothesis that the fringe is created by any surface light-confinement mechanism including waveguided photons. Finite element simulations further showed that the fringe period (439 nm) matches with the experimental measurements (442 ± 230 nm) (Fig. S17d). In summary, by varying either the excitonic emitter or substrate, we can observe formation of standing wave fringe patterns in the near-field. We, therefore, conclude that these fringe patterns are a universal near-field phenomenon when a nanoscale emitter is placed on a highly smooth and index mismatched substrate.

We report a comprehensive study on the near-field interaction of a plasmonic tip with localized nanoscale excitonic emitters by spatially imaging their emission patterns using tip-enhanced nano-spectroscopy. Taking advantage of nanoscale spatial-resolution capability of the tip, sub-wavelength interference of in-plane propagating electromagnetic modes can be analyzed under tapping mode operation. Hyperspectral maps clearly illustrate that photons from localized emitters can be emitted in-plane that can be visualized ~1.7 microns away from the emitting source by virtue of standing waves formation. The interference period and the corresponding signal decay rate is governed by dielectric layer thickness and permittivity which also dictates the fraction of photons radiated in plane and the degree of confinement. The exciton transition dipole orientation and presence of the tip determines shape and directionality of the standing wave. Strain-induced formation of localized emitters in a 2D dielectric medium is favorable in terms of in-plane radiation coupling efficiency. Finally, the difference in excitation energy and exciton emission energy is an important metric that dictates the mechanism involved in fringe formation. Our work shows that near-field scanning probe microscopy with metallic tips is a useful tool in imaging and analysis of nanoscale excitonic emitters including their radiation patterns as well as dipole orientations. In addition, our work helps understand energy transfer mechanisms and dynamics of excited state phenomena in emitters at deep sub-wavelength scales with both spectral and spatial information. The technique and approach could therefore serve as a useful tool for imaging, identifying and manipulating dipoles of even quantum emitters opening new avenues in classical and quantum nanophotonics.

## Methods

### Synthesis of CdSe nanoplatelets

Cadmium myristate precursor is prepared by following the literature^51^. Colloidal, rectangular CdSe nanoplatelets with a thickness of 4.5 monolayers are synthesized following the literature^52^ with slight modifications^53^.

340 mg of finely-ground cadmium myristate and 28 mL 1-octadecene (technical grade, ODE) are added to a 100 mL three-necked round-bottom flask with a 1-inch octagonal stir bar. The central neck is connected to the Schlenk line through a 100 mL bump trap, one of the side necks is equipped with a thermocouple adapter and thermocouple, and the other one is fitted with a rubber stopper. With a heating mantle, the flask is degassed at 100 °C for 30 min. In the meanwhile, a dispersion of 0.15 M selenium in ODE is prepared by sonication for at least 20 min. After switching the atmosphere of the flask to nitrogen, the temperature of the reaction is increased to 220 °C. 2 mL of 0.15 M Se/ODE dispersion are quickly injected by using a 22 mL plastic syringe equipped with a 16 G needle. After 20 s, 120 mg of finely ground cadmium acetate are added to the flask by temporarily removing the stopper. The flask is carefully rocked to ensure that the cadmium acetate powder does not stick to the side walls of the flask. The reaction is kept at 220 °C for 14 min and then rapidly cooled with a water bath. 12 mL of oleic acid (technical grade, OA) and 22 mL of hexane are added when the temperature reaches 160 °C and 70 °C, respectively.

### Washing procedure of CdSe nanoplatelets

The nanoplatelets are washed by following a procedure reported in the literature with modifications^54^. The mixture is first centrifuged at 8586 *g* for 10 min. The precipitate is then redispersed in 10 mL of hexane. The suspension is left undisturbed for 1 h, and then centrifuged at 6574 *g* for 7 min. The precipitate is discarded as it contains undesired 3.5 monolayer nanoplatelets. The supernatant is retained and transferred to a new centrifuge tube. 10 mL of methyl acetate are added to the supernatant, followed by centrifugation at 5668 *g* for 10 min. 6 mL hexane is used to redisperse the precipitate. Measuring the optical absorption spectrum is useful to confirm the removal of the unwanted 3.5 monolayer nanoplatelets, which are characterized by a lowest-energy absorption peak at 462 nm, while the 4.5 monolayer nanoplatelets are characterized by a lowest-energy absorption peak at 512 nm. If 3.5 monolayer nanoplatelets are still present in the dispersion, they can be removed by titrating methyl acetate and centrifuging until all 3.5 monolayer nanoplatelets are successfully removed. The final dispersion is stored in a glass vial in the dark.

### Growth of Cd_x_Zn_1-x_S shell

Cadmium oleate (Cd(Ol)_2_) and zinc oleate (Zn(Ol)_2_) are synthesized according to the literature^54,55^. The growth of Cd_x_Zn_1-x_S shell on CdSe nanoplatelets is performed by following the literature^54^ with minor modifications.

10 mL of ODE, 0.4 mL of OA, 90 mg of cadmium oleate, 167.5 mg of zinc oleate, and an amount of 4.5 monolayer CdSe nanoplatelets in hexane equivalent to a 1 mL with an optical density of 120/cm at the lowest-energy absorption peak are added to a 100 mL three-necked round-bottom flask with a 1-inch octagonal stir bar. The central neck is connected to the Schlenk line through a 100 mL bump trap, one of the side necks is equipped with a thermocouple adapter and thermocouple, and the other one is fitted with a rubber stopper. The mixture is degassed for 35 min at room temperature and for 15 min at 80 °C. In the meanwhile, a solution of 83 μL of 1-octanethiol (OT) in 7 mL of degassed ODE and 2 mL of degassed OA is prepared in the glovebox and loaded in a plastic syringe. 2 mL of degassed oleylamine are added to a second plastic syringe. The two syringes are removed from the glovebox. Afterward, the atmosphere of the reaction flask is switched to nitrogen and the 2 mL of OAm are injected. Using a heating mantle, the temperature of the reaction flask is increased to 300 °C. At 165 °C, the solution of OT in ODE and OA is injected at a rate of 4.5 mL/h. After complete injection, the temperature of the reaction is maintained for an additional 40 min. The reaction mixture is cooled down to 240 °C by using an air gun, followed by using a water bath to cool to room temperature. At 40 °C, 5 mL of hexane is added.

### Washing procedure of CdSe/Cd_x_Zn_1-x_S nanoplatelets

The reaction mixture is centrifuged at 6000 *g* for 6 min. The precipitate is redispersed in 5 mL of hexane while the supernatant is discarded. Methyl acetate is added to the dispersion until the mixture turns turbid, followed by centrifugation at 6000 *g* for 10 min. This process is repeated. The precipitate is redispersed in 3 mL of hexane and centrifuged at 6000 *g* for 7 min. The precipitate is discarded, containing aggregated nanoplatelets. The supernatant is retained and filtered through a 0.2 µm PVDF or PTFE syringe filter. The final dispersion is stored in a glass vial under ambient conditions in the dark.

### TEM imaging

For low-resolution TEM, a JEOL 1400 microscope was operated at 120 kV. For higher-resolution TEM, a JEOL F200 microscope was operated at 200 kV. During imaging, magnification, focus, and tilt angle were varied to yield information about the crystal structure and super structure of the particle systems. To prepare the dispersed nanocrystals for imaging, we drop cast 10 μL of a dilute (~0.1 mg/mL) dispersion of nanocrystals in hexane on a carbon-coated TEM grid (EMS). The grid was dried under vacuum for 1 h prior to imaging.

### Spectrophotometry

Absorption spectra of nanocrystal dispersions in toluene were measured by using a Cary 5000 UV-Vis-NIR spectrophotometer.

### Photoluminescence quantum yield

Photoluminescence quantum yield (PLQY) measurements were performed by using the integrating sphere module of an Edinburgh FLS1000 Photoluminescence Spectrometer. The NCs were dispersed at a concentration corresponding to an absorbance of 0.1 at the excitation wavelength.

### Dielectric-NP on Au sample preparation

The diluted CdSe/Cd_x_Zn_1-x_S nanoplatelet dispersion (0.001 mg/ml in toluene) was spin-coated on the template-stripped Au substrate. Template stripped Au substrate was used for exceptionally low rms value (0.5 nm)^56^. Dielectric layer (Al_2_O_3_, TiO_2_) were deposited by atomic layer deposition (Cambridge Nanotech S200 ALD). Refractive index of dielectric layers was measured by Ellipsometer (Woollam VAS Ellipsometer).

### Tip-enhanced photoluminescence imaging with contact and tapping mode

LabRam-EVO Raman/far-field PL Spectrometer (Horiba Scientific) coupled with AFM setup (OmegaScope-R, AIST-NT) was used to conduct tip-enhanced photoluminescence measurement. After 633 nm laser was aligned to the apex of the tip, the sample is engaged with the frequency-modulated feedback loop to measure the topography. Corresponding TEPL spectrum was obtained by both the contact and the tapping mode simultaneously. Each pixels in the hyperspectral map span 30 × 30 nm^2^ and signals were collected for 100 ms. Near-field TEPL map and spectrum were extracted by subtracting the contact mode TEPL to the tapping mode TEPL.

### Reporting summary

Further information on research design is available in the Nature Portfolio Reporting Summary linked to this article.

## Supplementary information

Supplementary Information

Reporting Summary

## Data Availability

The data that support the conclusions of this study are available from the corresponding author on request.
