# Supplementary material for: Direct nano-imaging of light-matter interactions in nanoscale excitonic emitters
Source: Nat Commun. 2023 May 8;14:2649. doi: 10.1038/s41467-023-38189-y (PMC10167231; doi:10.1038/s41467-023-38189-y)
Supplement: Supplementary file 1 — Supplementary Information [file 41467_2023_38189_MOESM1_ESM.pdf]

Supporting Information

Direct Nano-Imaging of Light-Matter Interactions in Nanoscale Excitonic Emitters

Kiyoung Jo[1], Emanuele Marino[2,3], Jason Lynch[1], Zhiqiao Jiang[2,4], Natalie Gogotsi[4], Thomas P. Darlington[5], Mohammad Soroush[6], P. James Schuck[5], Nicholas J. Borys[6], Christopher B. Murray[2,4], Deep Jariwala[1]*

[1]Department of Electrical and Systems Engineering, University of Pennsylvania, PA, 19104, United States

[2]Department of Chemistry, University of Pennsylvania, PA, 19104, United States

[3]Dipartimento di Fisica e Chimica, Università degli Studi di Palermo, Via Archirafi 36, 90123 Palermo, Italy

[4]Department of Materials Science and Engineering, University of Pennsylvania, PA, 19104, United States

[5]Department of Mechanical Engineering, Columbia University, New York, New York, 10027, United States

[6]Departement of Physics, Montana State University, Bozeman, Montana, 59717, United States

*Corresponding author: dmj@seas.upenn.edu

Analysis

A. Tip-enhanced photoluminescence imaging with contact and tapping mode

The tapping mode and contact mode operation has been determined by simultaneous amplitude and force curve vs piezo-displacement (Figure S1a). The tapping mode operation maintains a few nN of attractive force while the amplitude is slightly reduced from the free amplitude as shown in Figure S1b. The snap to contact occurs at a piezo displacement of -18 nm and the amplitude becomes approximately 100, which is very far from our tapping mode condition. Therefore, we believe that the tip does not directly contact the sample during the tapping mode operation. For contact mode, we applied a repulsive force of 12 nN to make contract. The resulting tip-sample distance at 12 nN was calculated to be 0.255 nm based on the Lennard-Jones model and taking into account the titanium dioxide (TiO_2) capping layer on the NPs as shown in Figure S1c. ($f = Ad^{-13}$ where $A = 2.87*10^{-7}$ nN nm^{13}).[1] The constant A was calculated by adopting the reported Lennard-Jones parameters for Au-O bond considering TiO_2 capping layer.[2] This distance is in the quantum tunneling/charge transfer regime, which can cause optical quenching according to Zhang et al.[1]. However, we did not observe any quenching behavior due to the presence of the TiO_2 capping layer.

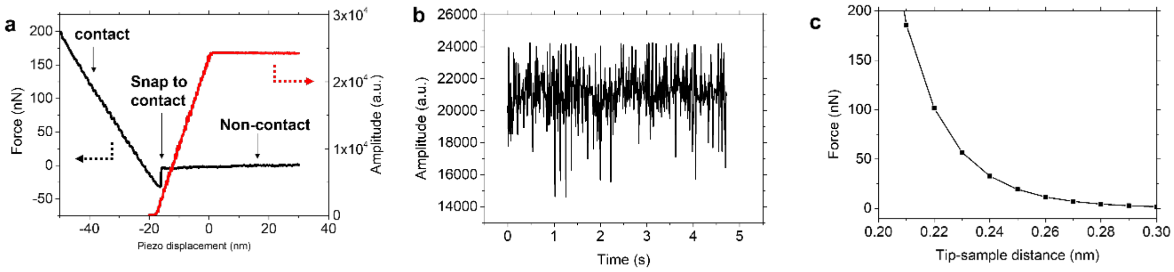

Figure S1. Tip-sample distance control. (a) Force (black) and tip amplitude (red) vs piezo displacement curve. (b) Tip amplitude vs time plot during the tapping mode operation. (c) Force vs tip-sample distance curve calculated by Lennard-Jones model.

B. Strong coupling between NP excitons and tip plasmons in contact mode TEPL

In tapping mode, the tip oscillates with a 20 nm amplitude[3] and is only a few nanometers away from the surface at its extrema in the oscillation. For this reason, tapping mode operation under frequency modulated feedback does not bring the tip close enough to the sample to achieve the large field enhancement originating from the plasmonic gap mode, precluding the formation of strongly coupled, hybridized light matter states such as exciton-polaritons. Conversely, in contact mode the tip-sample distance is < 1 nm. Subsequently, either plasmonic gap- or tip-mode triggers strong coupling between the excitons of the emitter and light unless close tip contact dive into the quantum regime and quenches the excitonic emission and Raman.[1] we investigate strong light-matter coupling between the quasi-2D CdSe-$Cd_xZnS_{1-x}S$ core-shell nanoplatelets (NPs) and the plasmonic tip collecting spectral data at each pixel with ~20 nm (tip radius) spatial resolution while concurrently mapping the topography. Topography images show that the NPs are aggregated to form 200 nm-wide clusters, which are likely resulting from the high dilution factor (Figure S2a). The thickness of the cluster varies between 4.9 and 50

nm, and the emission spectra varies drastically as a function of the cluster thickness (Figure S2a, b). This local variation of the TEPL spectrum is evident in Figure 1d. For a 40 nm thick NP cluster, a single emission at 664 nm was observed reflecting the excitonic emission of the NP (Figures S2c, d). On the other hand, a 4.9 nm thick cluster, corresponding to the thickness of a single NP with ligand showed an additional feature at 699 nm, as shown comparison between normalized far-field and contact-mode TEPL spectrum (Figures S2c, d, e). This feature is observed neither from NPs dispersed in solution nor from the NPs under electrical bias (Figure S3). Therefore, we attribute it to the lower exciton-polaritonic emission. The peak splitting has been reported in plasmonic cavity.[4,5] However, in our system, Upper polariton branches do not show up because it is filtered out by edge-filter mounted in front of detector to block laser.

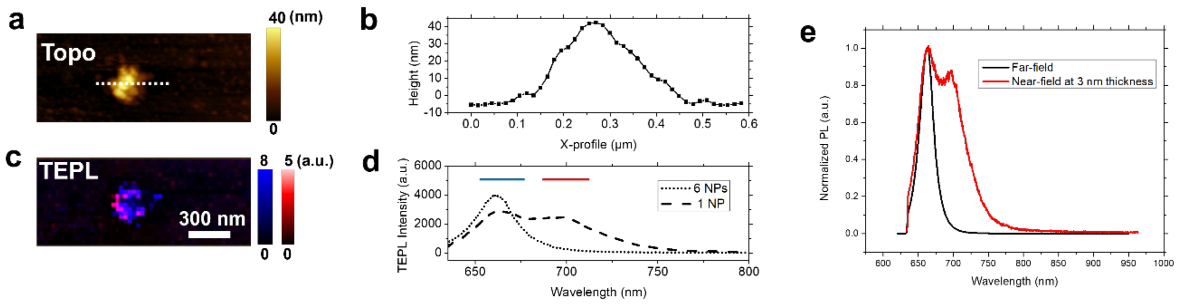

Figure S2. Optical characterization under contact mode TEPL. (a) Topography and (b) height profile along dotted line in (a). (c) Hyperspectral TEPL map under contact mode and (d) spectrum at two different NP thickness. Solid color line represents spectral window of its color in (c). (e) Normalized far-field PL intensity (black) and the contact mode TEPL (red).

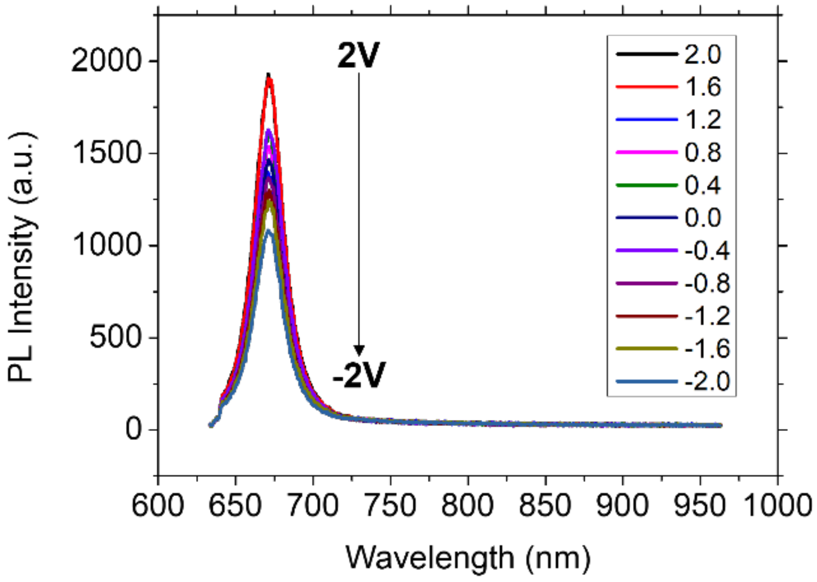

Figure S3. TEPL with contact mode tip under bias with different voltage on TiO_2/NP/Au. 13 nm thick NP cluster is used for the measurement.

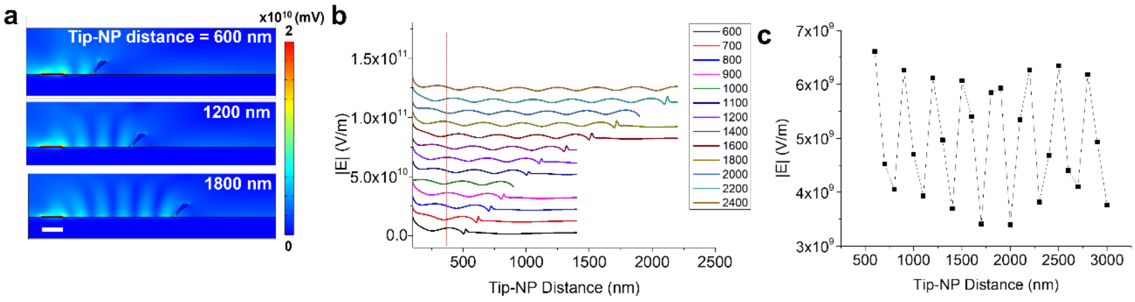

Figure S4. Simulation about fringes with respect to different tip-NP distances. (a) Simulated 2D map of E field fringes between NP and tip with respect to tip-NP distance. (b) Extracted E-field strength profile at z = 10 nm as a function of distance. (c) E-field strength at the fixed point where 400 nm away from NP. Scale bar in (a) represents 300 nm.

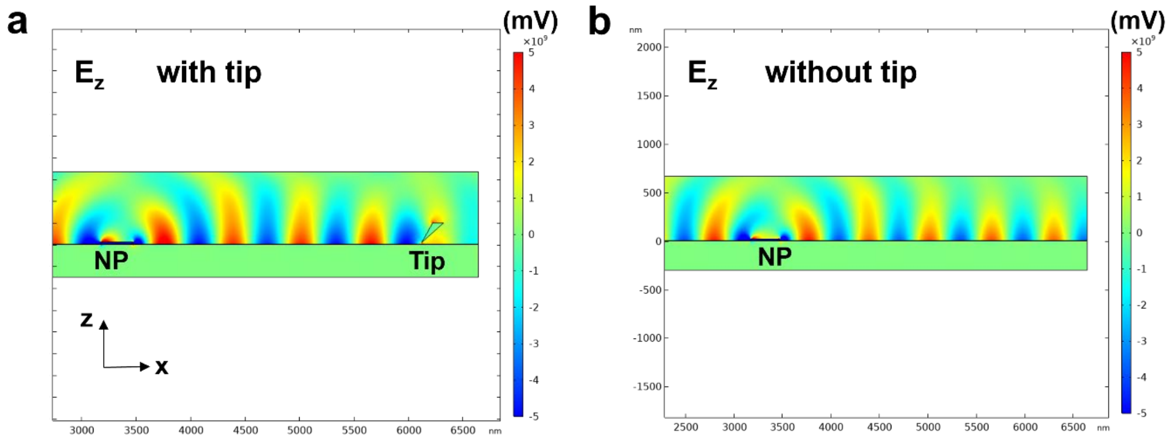

Figure S5. z-component electric field map. (a, b) E_z field map of TM polarized NP emission on Au (a) with and (b) without the tip.

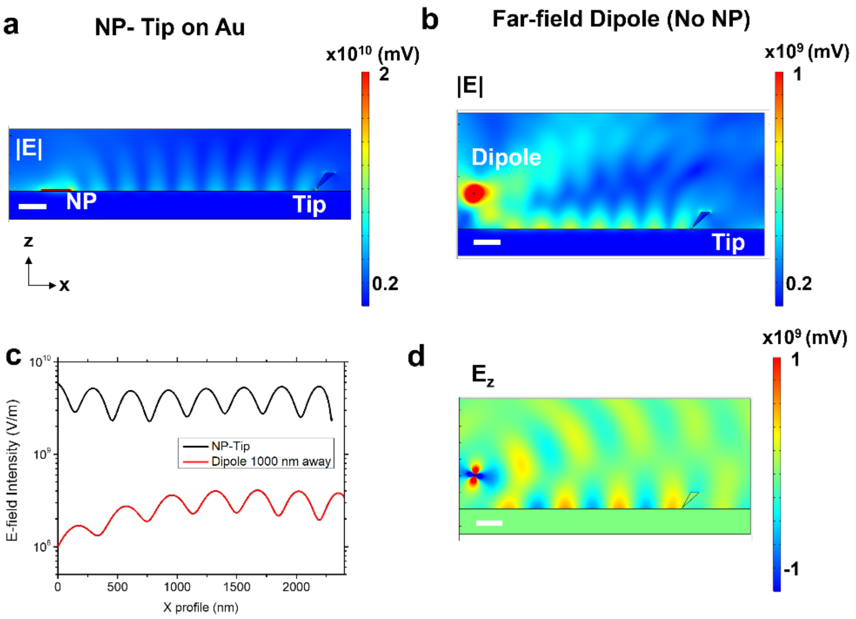

Figure S6. Dipole away from plasmonic substrate. (a, b) E-field strength map of (a) Al_2O_3 (5 nm)/NP/Au with tip system and (b) Al_2O_3 (5 nm)/Au with tip and dipole 1000 nm away from the surface without NP. (c) Comparison of E-field intensity of (a) and (b) at z = 10 nm. (d) E_z field profile of Al_2O_3 (5 nm)/Au with tip and dipole 1000 nm away from the surface without NP. Scale bars in (a), (b) and (d) indicate 300 nm.

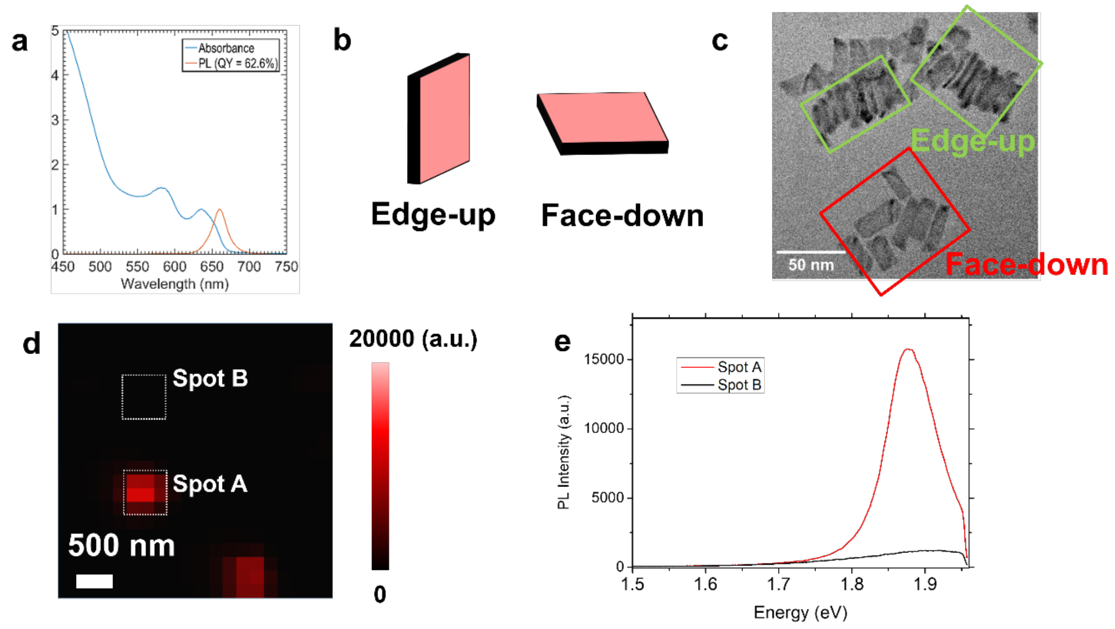

Figure S7. Optical properties of NPs. (a) Absorption and emission spectra of NP solution. Photoluminescence quantum yield (PLQY) is indicated in the figure legend. (b) Schematic representation of edge-up assembled and face-down assembled NP cluster. (c) TEM image of NP clusters. (d) Far-field PL map of TiO_2/NP on Au using conventional far-field setup using 633 nm excitation laser and (e) PL spectrum corresponding to the spots indicated in (d). Pixel size is 200*200 nm^2.

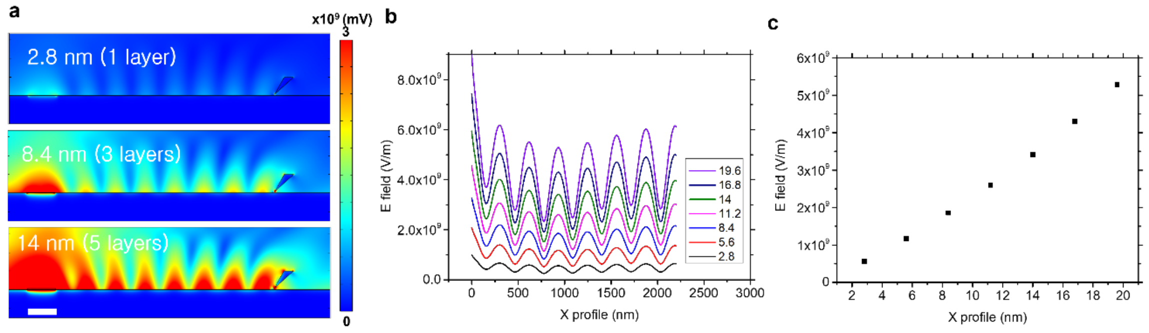

Figure S8. E-field maps as a function of NP thicknesses. (a) E-field map with different NP thickness. (b) E-field along x-direction at height =20 nm (c) E-field intensity vs NP thickness at node. Scale bar in (a) represents 300 nm.

C. Determination of fringe period from hyperspectral images

Discrete Fourier Transformation was performed to determine the fringe period.[6] TEPL linecut profiles (Intensity vs x) were conducted by analyzing the data with eqn. (1) below.

$$F_n = \sum_{i=0}^{N-1} x_i e^{-\frac{2\pi j}{N} ni} \qquad (1)$$

See Figure S9 to find the FFT plots.

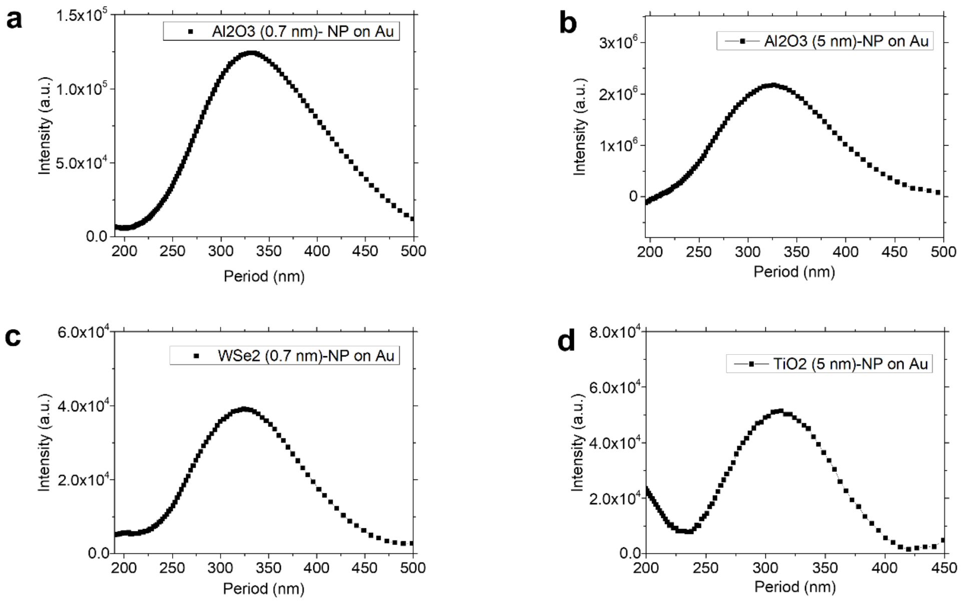

Figure S9. Determination of fringe period. Fourier transformed periodic fringe profiles extracted from the near-field TEPL maps for (a) Al_2O_3 (0.7 nm)/NP/Au (b) Al_2O_3 (5 nm)/NP/Au (c) WSe_2 (0.7 nm)/NP on Au and (d) TiO_2 (5nm)/NP/Au.

D. Decaying constant

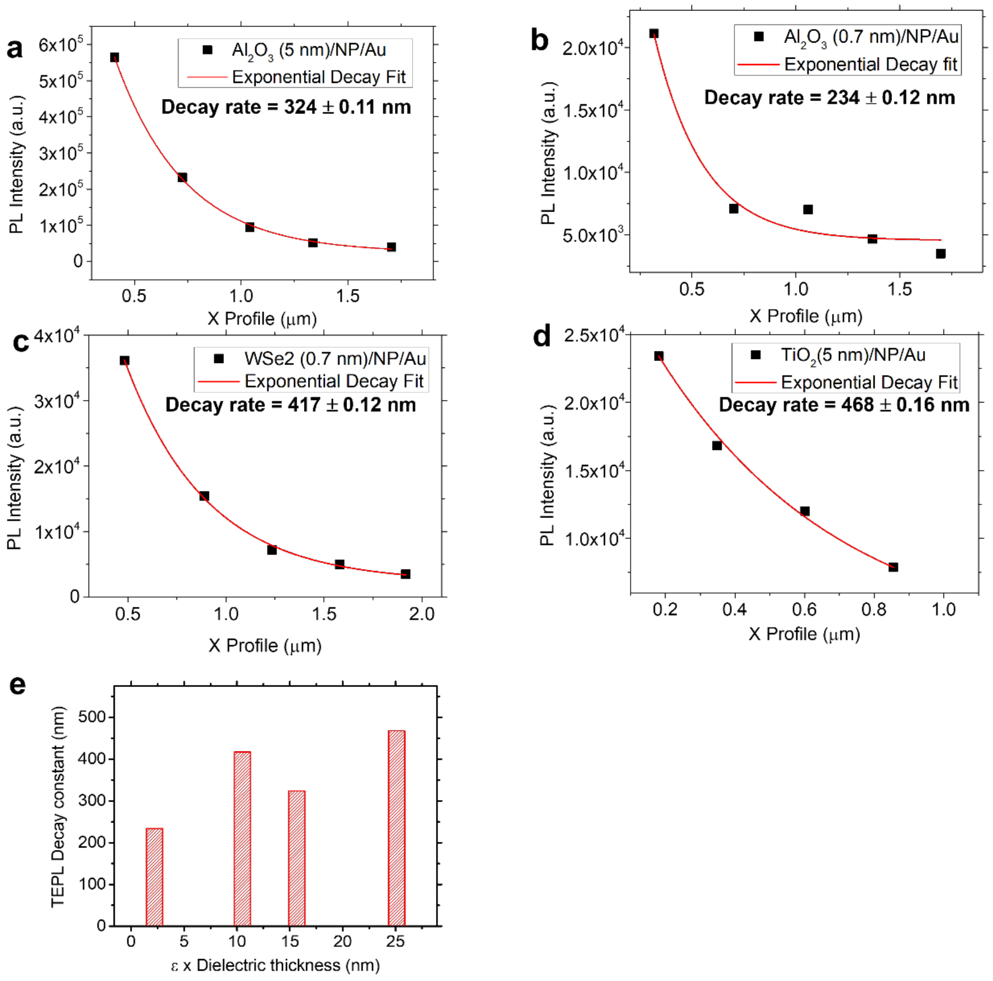

Figure S10. Decay constant of SPPs. Tapping mode TEPL intensity at antinodes of the fringes of (a) Al_2O_3 (5 nm)/NP/Au (b) Al_2O_3 (0.7 nm)/NP/Au (c) WSe_2 (0.7 nm)/NP/Au (d) TiO_2 (5 nm)/NP/Au (e) TEPL intensity decay constants from exponential decay fit. The fringe period was plotted as a function of the product of dielectric permittivity (ε) and thickness.

Decay constants (β) were obtained by fitting exponential decay curve with eqn. (2) below.

$$y = y_o + A_o e^{-\frac{x - x_o}{\beta}} \qquad (2)$$

The exponential decay fittings were iterated 500 times with a tolerance of $1*10^{-15}$.

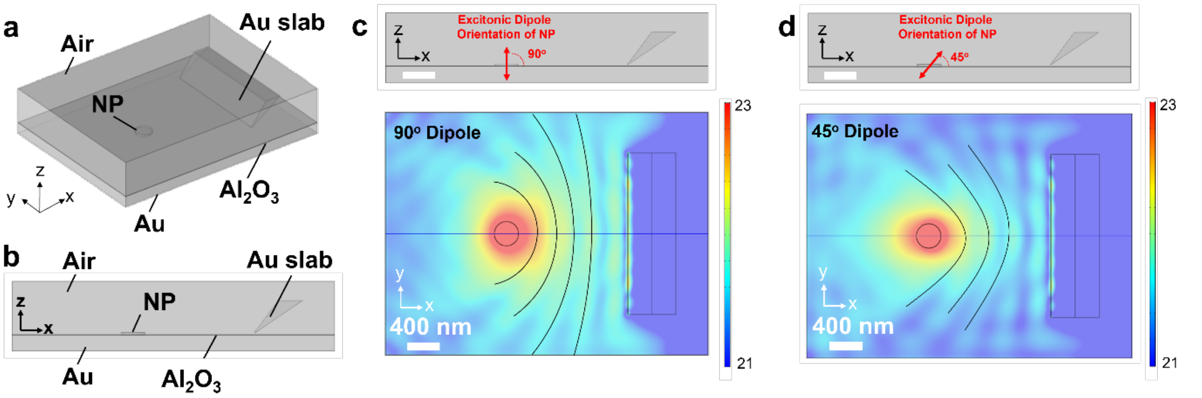

Figure S11. 3D simulation with different dipole orientation. (a, b) Schematic representation of Al_2O_3 (5 nm)/NP/Au with Au Slab with (a) isometric view and (b) side view used for the 3D simulations. Au slab represents the trajectory of Au tip during the scan. (c, d) Side view with excitonic dipole orientation (top) and in-plane E-field strength map at z = 5 nm above the dielectric layer (bottom) with (c) 90° (z-directional) excitonic dipole (d) 45° angle of excitonic dipole in XZ plane with respect to the sample plane. Scale bars indicate 400 nm.

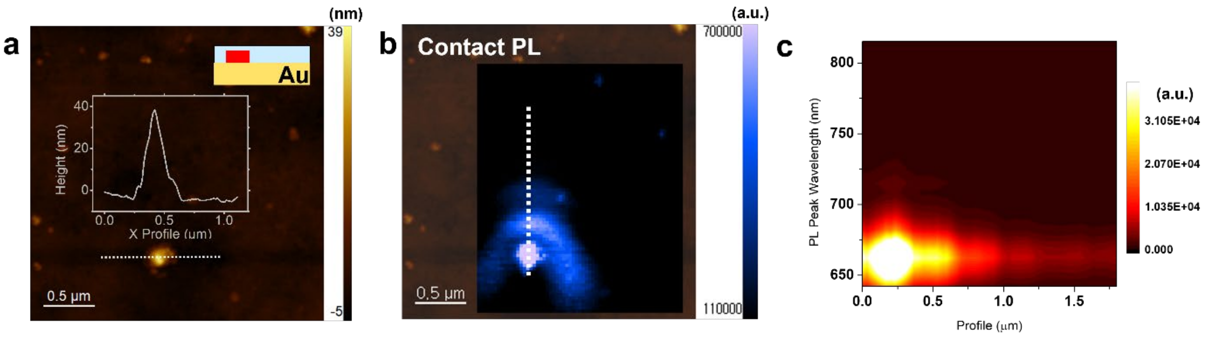

Figure S12. Contact mode hyperspectral map. (a) Topography image of Al_2O_3 (5 nm)/NP/Au and (b) contact mode TEPL at 664 nm (c) TEPL intensity Profile along dotted line in (b) with respect to PL peak wavelength. Inset in (a) indicates the height profile along the dotted line in (a).

E. Analytical Model for E-K Diagram of Surface Plasmon Polariton

Lorentz-Drude model is adopted to describe dielectric function of Au as shown in eqn. (3) below.

$$\epsilon_m(\omega) = 1 + \sum_j \frac{f_j \omega_p{}^2}{\omega_o{}^2 - \omega^2 + i\Gamma_j \omega} \qquad (3)$$

where f_j is oscillator strength, ω_p is plasma frequency, ω_o is oscillator resonance frequency, Γ_j is damping ratio. Parameters are taken from previous report.[7]

The term at j=1 is the Drude model with $\omega_o = 0$, describing metal dispersion under low frequency. Other terms represent the interband transition of the Au, which affects from ~1.8 eV due to large Gaussian broadening of the interband transition at ~2 eV.[7] The dielectric function of other dielectric layers are experimentally obtained by ellipsometry. E-K diagram is simulated by using finite-element method simulation.

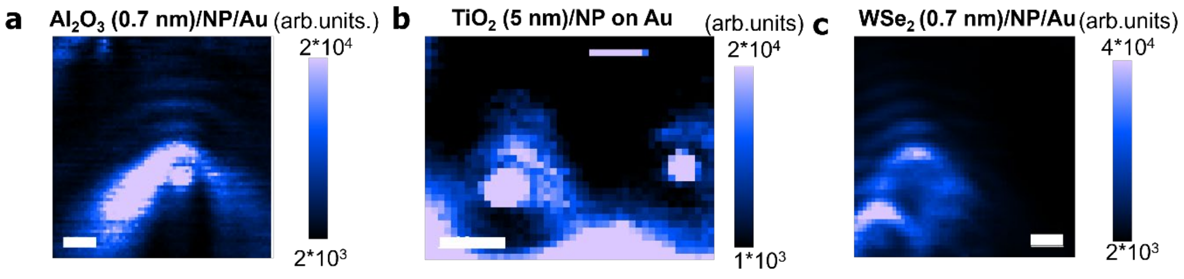

Figure S13. TEPL maps with different dielectric materials. Hyperspectral TEPL intensity map (λ=664 nm) of (a) Al_2O_3 (5 nm)/NP/Au and (b) TiO_2 (5 nm)/NP/Au and (c) WSe_2 (0.7 nm)/NP/Au. Scale bars indicate 500 nm.

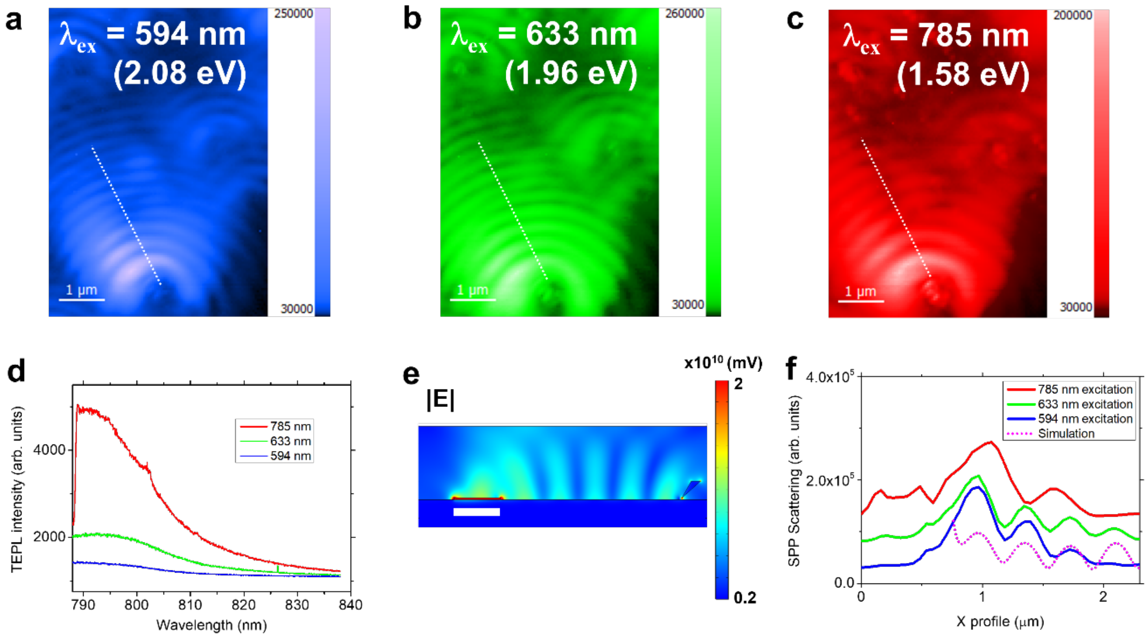

Figure S14. Fringes with different excitation wavelengths. (a-c) Hyperspectral map of tip-enhanced photoluminescence from WSe_2 nanobubbles on Au. The spectral window for the maps is 1.55 – 1.57 eV (790 -800 nm) under (a) 2.08 eV (b) 1.96 eV and (c) 1.58 eV excitation laser. (d) Tip-enhanced photoluminescence spectrum of WSe_2 nanobubble corresponding to the maps in (a)-(c). (e) Simulated E-field profile of dipole emitter at 790 nm in proximity of Au surface with WSe_2 dielectric surrounding. (f) Comparison of experimentally obtained fringe profiles along dotted line in (a-c) and the simulated fringe profile. Electric dipole emitting at 790 nm is placed in vicinity of Au substrate for the simulation. Scale bars in (a)-(c) represents 1 μm and scale bar in (e) indicates 300 nm.

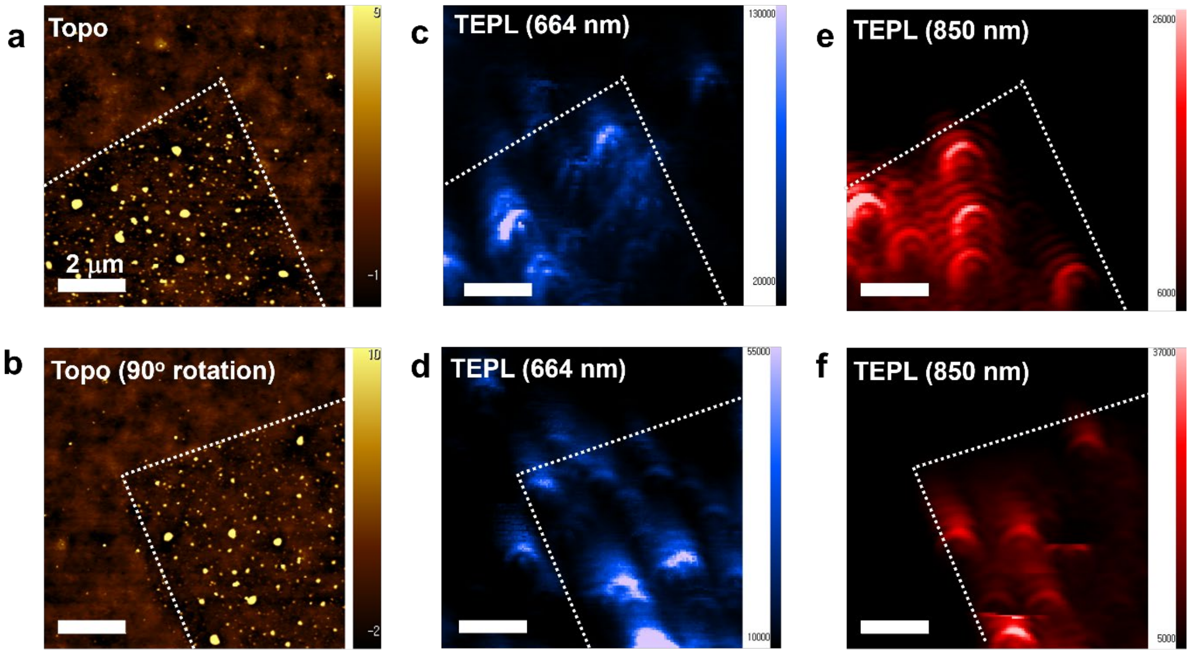

Figure S15. Fringes before and after sample rotation. (a, b) Topography image of Al_2O_3/NP/WSe_2/Au (a) before and (b) after the 90° rotation. (c, d) The emission from the NP (c) before and (d) after the 90° rotation. (e, f) The emission from the WSe_2 nanobubbles (c) before and (d) after the 90° rotation. The fringe direction does not change upon rotation of sample as seen in (c-f) Scale bars indicate 2 μm.

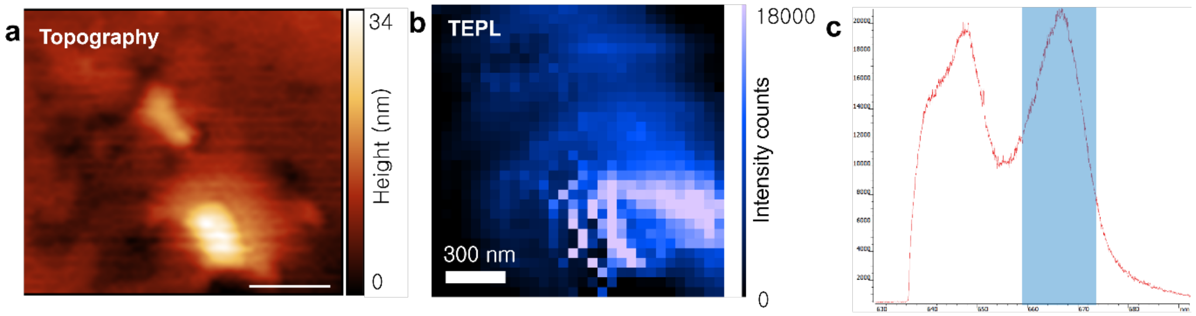

Figure S16. Fringes from WS_2 nanobubble. (a) Topography, (b) the contact mode TEPL map and (c) corresponding spectrum of WS_2 nanobubble.

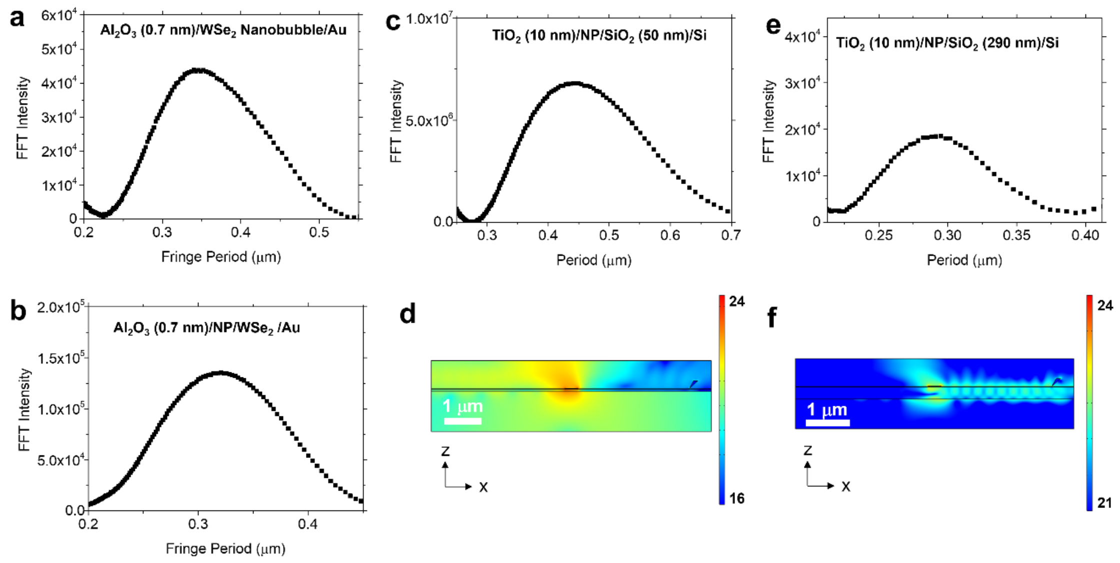

Figure S17. Fringe period determination of different systems. Fourier transformed periodic fringe profiles extracted from the near field TEPL maps for (a) Al_2O_3 (0.7 nm)/WSe_2 nanobubbles/Au and (b) Al_2O_3 (5 nm)/NP/WSe_2/Au. (c, d, e, f) Fourier transformed periodic fringe profiles extracted from the near field TEPL maps for (c) TiO_2 (10 nm)/NP/SiO_2 (50 nm)/Si and (e) TiO_2 (10 nm)/NP/SiO_2 (290 nm)/Si. Simulated E-field strength map of (d) TiO_2 (10 nm)/NP/SiO_2 (50 nm)/Si and (f) TiO_2 (10 nm)/NP/SiO_2 (290 nm)/Si.

Supplementary References

1. Zhang, Y. *et al.* Improving resolution in quantum subnanometre-gap tip-enhanced Raman nanoimaging. *Sci. Rep.* **6**, 25788 (2016).
2. Hu, H. & Sun, Y. Molecular dynamics simulations of disjoining pressure effect in ultra-thin water film on a metal surface. *Appl. Phys. Lett.* **103**, 263110 (2013).
3. Chen, J. *et al.* Optical nano-imaging of gate-tunable graphene plasmons. *Nature* **487**, 77–81 (2012).
4. Park, K.-D. *et al.* Tip-enhanced strong coupling spectroscopy, imaging, and control of a single quantum emitter. *Sci. Adv.* **5**, eaav5931 (2019).
5. Chikkaraddy, R. *et al.* Single-molecule strong coupling at room temperature in plasmonic nanocavities. *Nature* **535**, 127–130 (2016).
6. Fei, Z. *et al.* Nano-optical imaging of WSe2 waveguide modes revealing light-exciton interactions. *Phys. Rev. B* **94**, 081402 (2016).
7. Rakić, A. D., Djurišić, A. B., Elazar, J. M. & Majewski, M. L. Optical properties of metallic films for vertical-cavity optoelectronic devices. *Appl. Opt.* **37**, 5271 (1998).
